# Drug Development in Alzheimer’s Disease: The Contribution of PET and SPECT

**DOI:** 10.3389/fphar.2016.00088

**Published:** 2016-03-31

**Authors:** Lieven D. Declercq, Rik Vandenberghe, Koen Van Laere, Alfons Verbruggen, Guy Bormans

**Affiliations:** ^1^Laboratory for Radiopharmacy, Department of Pharmaceutical and Pharmacological Sciences, KU LeuvenLeuven, Belgium; ^2^Laboratory for Cognitive Neurology, Department of Neurosciences, KU LeuvenLeuven, Belgium; ^3^Nuclear Medicine and Molecular Imaging, Department of Imaging and Pathology, KU LeuvenLeuven, Belgium

**Keywords:** Alzheimer’s disease, PET, SPECT, drug development, biomarkers

## Abstract

Clinical trials aiming to develop disease-altering drugs for Alzheimer’s disease (AD), a neurodegenerative disorder with devastating consequences, are failing at an alarming rate. Poorly defined inclusion-and outcome criteria, due to a limited amount of objective biomarkers, is one of the major concerns. Non-invasive molecular imaging techniques, positron emission tomography and single photon emission (computed) tomography (PET and SPE(C)T), allow visualization and quantification of a wide variety of (patho)physiological processes and allow early (differential) diagnosis in many disorders. PET and SPECT have the ability to provide biomarkers that permit spatial assessment of pathophysiological molecular changes and therefore objectively evaluate and follow up therapeutic response, especially in the brain. A number of specific PET/SPECT biomarkers used in support of emerging clinical therapies in AD are discussed in this review.

## Introduction

The worldwide prevalence of AD is estimated at 35 million, a number expected to quadruple by 2050, due to the increasing lifespan of the world population (Brookmeyer et al., 2007). With an unfavorable prognosis and a life expectancy of approximately 8–10 years, AD is becoming one of the most costly diseases for society (Thies and Bleiler, 2013). In spite of increasing knowledge about the genetics, epidemiology, and histopathological features of AD there is, at this moment, only symptomatic treatment available. However, already at early clinical signs, intrinsic disease progression has developed for a long time; patients rapidly decline and develop irrevocable brain damage (Masters et al., 2006; Romano and Buratti, 2013). Therefore, there is a great need for efficient treatment that should be initiated in a very early phase of the disease. Strict definite diagnosis can still only be made post-mortem, on the basis of two pathological hallmarks: SP and NFTs (Hyman et al., 2012), although use of biomarkers is strongly advocated in research guidelines (Dubois et al., 2014). More than 90% of clinical trials aiming to intervene at the causative pathological elements have failed to produce disease altering effects. A major concern hereby is the lack of objective biomarkers assisting in the evaluation of inclusion- and outcome criteria of participating patients, as many participants turned out to be misdiagnosed, particularly in the early AD disease stages (Jack et al., 2010; Barthel et al., 2015; James et al., 2015). Currently five biomarkers for AD have been used for evaluation of disease and monitoring of disease progression (Jack et al., 2010): CSF levels of Aβ_42_, CSF levels of total tau and p-tau_181_ and p-tau_231_, structural imaging (CT and MRI) and functional imaging (PET with [^18^F]FDG). Elevated levels of CSF tau and reduced levels of CSF Aβ allow the prediction of, respectively, the NFT load and the SP deposits (Ahmed et al., 2014; de Souza et al., 2014; Wurtman, 2015). Therefore, these CSF levels appear to be useful biomarkers in the diagnosis of AD (Wallin et al., 2006; Weiner et al., 2015). Nonetheless, CSF sampling requires an invasive lumbar puncture, quantification of CSF levels is hampered by interlaboratory variability and CSF values do not provide regional information on tau- and Aβ deposits. The regional concentration of tau- and Aβ deposits is however essential for a differential diagnosis of AD, especially among the different tauopathies (Hampel and Teipel, 2004; Gozes et al., 2009; Hampel et al., 2010). Structural volume measurement can be used to measure regional cerebral atrophy. Although not highly specific for AD due to overlap with ‘normal’ aging, the degree of atrophy follows neuropathological progression in AD and severity of volume loss correlates well with disease progression (Masters et al., 2006; Jack et al., 2010). Regarding the biomarkers that can be visualized and quantified by the molecular imaging techniques PET and SPECT, there are three important applications to be considered, that could contribute to successful drug development in clinical trials. The first one is the ability of PET and SPECT to provide quantitative and spatial *in vivo* assessment of, for example, the amyloid- and tau burden in AD patients. By doing so, inclusion and exclusion criteria in clinical trials can be verified more objectively than what was possible now with the previous biomarkers. Indeed, the use of specific radiotracers, for various targets, may provide accurate differential diagnosis (even at early AD stages) and true confirmation of the availability of the drug target, which allows physicians to reliably select patients for clinical trials to evaluate novel AD therapeutics (Barthel et al., 2015). Another important role for these molecular imaging techniques is the assessment and quantitative follow-up of drugs aiming to intervene at the specific molecular pathophysiological processes. Using highly selective PET- or SPECT radioligands, the true biological effect of novel clinical candidates can be established and true quantitative assessment is possible (Barthel et al., 2015). Thirdly, molecular imaging can be applied to measure the dose-related occupancy of specific targets caused by drugs under test, which allows the characterization of the optimal therapeutic window and thus a more effective design of subsequent clinical drug trials (Broich et al., 1998; Passchier et al., 2002). Although PET is able to provide a much higher spatial resolution and dynamic scanning with higher temporal resolution and better quantification than SPECT, SPECT cameras are more widely available and cheaper than PET cameras. Both the availability and the economical aspect are important to consider when performing large multi-center clinical trials (Rahmim and Zaidi, 2008).

**FIGURE 1:**
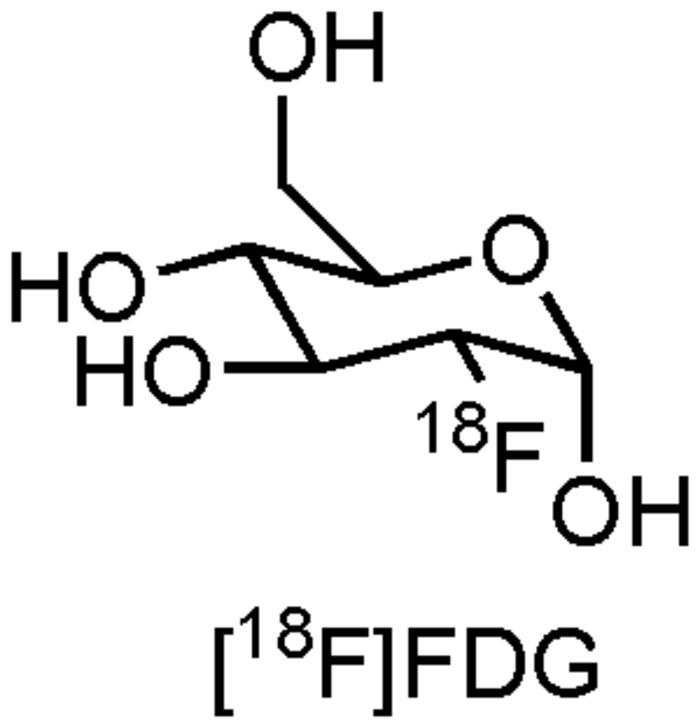
**Structure of [^18^F]FDG**.

The most frequently used PET radiopharmaceutical is 2-[^18^F]fluoro-2-deoxy-D-glucose ([^18^F]FDG, **Figure 1**), a glucose derivative which allows measurement of brain glucose metabolism directly related with viability of brain tissue in AD (Barthel et al., 2015). This commercially available compound, with various clinical applications, has been well established in routine clinical practice, but also in the recruitment and follow-up of the majority of AD clinical trials which use PET as biomarker technique (Barthel et al., 2015). Indeed, several AD clinical trials are currently recruiting and following up patients with [^18^F]FDG (CTI: NCT02593318, NCT01561053, and NCT02560753). Although FDG-PET is able to provide information about regional glucose metabolism, which can aid in the detection and prognosis of MCI for further progression to AD, there is a great need for PET- and SPECT radiopharmaceuticals which deliver more target-specific information of a variety of (patho)physiological processes that are happening in AD. In this review we will therefore focus on some of the major molecular pathophysiological changes known to occur in AD, along with emerging pharmacological treatment approaches. Furthermore, specific attention will be attributed to the role that PET- and SPECT biomarkers (can) play during these clinical trials.

## Cholinergic Hypothesis

### Acetylcholinesterase

The cholinergic hypothesis states that a decreased cholinergic neurotransmission, caused by a degeneration of cholinergic neurons in the basal forebrain, leads to several cognitive and functional conditions, associated with the symptoms of AD (Davies and Maloney, 1976; Bartus et al., 1982). Furthermore, the disruption of AChE seems to be associated with NFT- and Aβ deposits (Tavitian et al., 1993).

Acetylcholine, a neurotransmitter synthesized presynaptically by ChAT, is released in the synaptic cavity, where it is able to interact with nicotinic and muscarinic cholinergic receptors on pre- and postsynaptic membranes. Synaptic transmission is eventually stopped by the hydrolysis of ACh by AChE (Lleo et al., 2006). Both ChAT and AChE expression is reduced in cortical regions of AD patients (Davies and Maloney, 1976; Coyle et al., 1983; Vogels et al., 1990).

Inhibition of AChE was the first approach to treat AD, and this led to the FDA approval of eventually four AChE inhibitors: galantamine, rivastigmine, donepezil, and tacrine, though the latter one was largely discontinued due to hepatotoxicity issues (Wu et al., 2010). All inhibitors showed however only mild symptomatic improvement in patients with mild to moderate AD (Burns et al., 1999; Rosler et al., 1999; Farlow et al., 2000; Greenberg et al., 2000; Raskind et al., 2000; Tariot et al., 2000; Wilcock et al., 2000; Winblad et al., 2001). Several new agents are currently under development (Aprahamian et al., 2013), two of which have been evaluated in clinical trials: phenserine (Winblad et al., 2010; Darreh-Shori et al., 2014) and huperzine A (Rafii et al., 2011). Phenserine, structurally related to rivastigmine, showed a prolonged, but mild inhibition of AChE in AD patients. Researchers suggested an add-on therapy with donepezil to improve the clinical efficacy of this class of agents. Low dosage of huperzine A, a reversible AChE inhibitor, showed no significant improvement on the ADAS-cog [the primary cognitive outcome measure in mild to moderate AD patients (259)], in a phase II trial in mild to moderate AD patients. Higher dosage and a long term evaluation were suggested by the authors. A phase III trial (CTI:NCT01282619) using sustained-release huperzine A is currently ongoing (Ghezzi et al., 2013).

**FIGURE 2:**
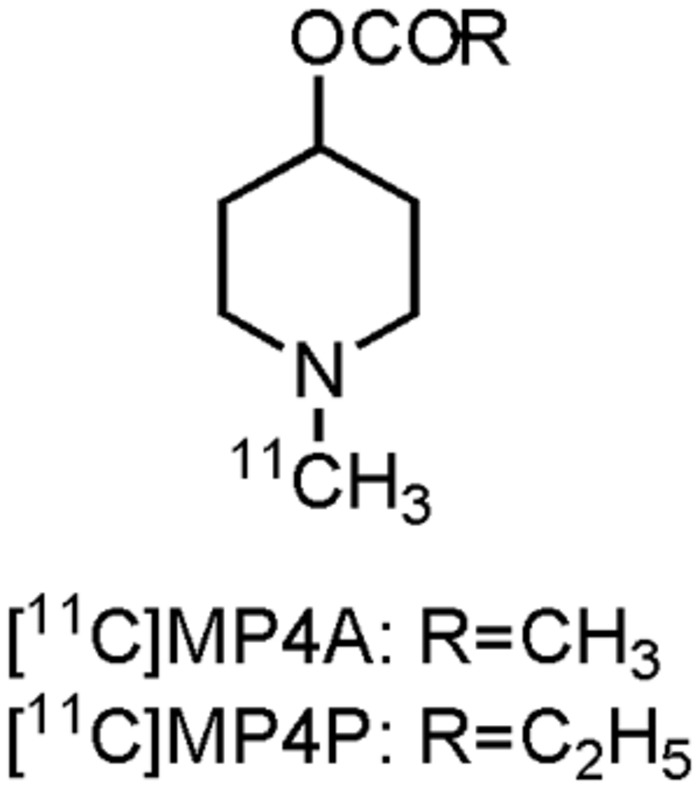
**Structure of acetylcholinesterase PET tracers**.

Visualization of the cholinergic system could be done by using radiolabeled ACh analogs or inhibitors of AChE (Tavitian et al., 1993; Irie et al., 1994; Iyo et al., 1997; Kilbourn et al., 1996). Both pathways have been pursued by researchers, but at present only two carbon-11 labeled compounds have been clinically evaluated on AD patients: [^11^C]MP4A and [^11^C]MP4P, two N-[^11^C]methylpiperidine esters (acetate and propionate, **Figure 2**). Moreover, both compounds were used to evaluate the effect of donepezil or rivastigmine in AD patients. Scans with [^11^C]MP4A or [^11^C]MP4P, taken before and after treatment with donepezil or rivastigmine, showed significant (up to 40%) cerebral cortical or frontal cortical inhibition of the AChE activity. Modest symptomatic improvement was recorded for all AD patients during these trials (Kuhl et al., 2000; Shinotoh et al., 2001; Kaasinen et al., 2002). These studies show the usefulness of both tracers for therapeutic monitoring of AChE inhibitors, as well as the possibility to evaluate newly developed drugs that target AChE (Shinotoh et al., 2004). Nonetheless, carbon-11 labeled compounds have the limitation that an on-site cyclotron is needed, which limits their widespread use. The lack of significant cognitive improvement and the fact that the cholinergic deficit is not an early event in the development of AD (Gilmor et al., 1999), has challenged the cholinergic hypothesis (Francis et al., 1999; Bartus, 2000; Terry and Buccafusco, 2003; Contestabile, 2011). Nevertheless, two decades after FDA approval of tacrine, AChE inhibitors remain (out of necessity) the mainstay for the current symptomatic treatment of AD.

### Muscarinic ACh Receptor

The presynaptic cholinergic signal is transmitted through the release of the neurotransmitter ACh, which can interact with both muscarinic and nicotinic ACh receptors (the latter one discussed in the next section). The pre-and postsynaptic muscarinic ACh receptor (mAChR) is a plasmamembrane, GTP binding protein coupled receptor, of which five subtypes exist (M_1-_M_5_) (Bonner et al., 1987; Peralta et al., 1987; Bonner, 1989). Due to the involvement in several neurological and psychiatric disorders, this target has been the topic of many research papers during the past few decades (Palacios et al., 1990; Tandon et al., 1991; Maziere, 1995). A comprehensive autoradiography study using tritium labeled compounds on the distribution of M_1_–M_4_ muscarinic receptors of histopathological diagnosed AD patients by Rodríguez-Puertas et al. (1997) showed that the decrease of M_1_ muscarinic receptors followed the general pattern of neurodegeneration as recorded in the Braak stages (Braak and Braak, 1991), while the M_2_ muscarinic receptor displayed significant reduction in the hippocampal area (up to 64%) and a significant increase in the striatum (up to 468% in the putamen), although this increase was not confirmed by other research groups (Eckelman, 2002). The density of M_3-4_ receptors on the other hand, was not altered compared to their density in brains of HCs. Interestingly, several research groups demonstrated that the stimulation of the M_1_- and M_3_ muscarinic receptors lead to an increase of the neuroprotective non-amyloidogenic pathway (formation of α-APP) (Buxbaum et al., 1992; Nitsch et al., 1992). Stimulation of these receptors could thus provide means for a decrease in Aβ production, a hypothesis which was confirmed in several studies (Wolf et al., 1995; Savonenko et al., 2005; Tsang et al., 2006). Little is known about the M_5_ muscarinic receptor, the latest receptor to be cloned (Bonner et al., 1988; Liao et al., 1989), although some research groups have demonstrated possible involvement in regulation of the cerebral blood flow and DA release (Yamada et al., 2001).

A number of clinical trials have been carried out to examine the potential role of mAChR agonists/antagonists on the clinical symptoms of AD patients. In the group of the selective M_1_ agonists, talsaclidine showed a significant decrease (up to 27% compared to placebo) in the Aβ-CSF levels in a randomized, double-blind, placebo controlled trial on AD patients with a MMSE score between 12 and 26. Results should, however, be interpreted with some caution, as suggested by the researchers, since assessment of the amyloid burden in AD patients by CSF has some flaws as biomarker, furthermore there was no mention on any cognitive improvement alongside the drop in Aβ CSF levels (Hock et al., 2000). Yet, another M_1_selective agonist, cevimeline (AF102B), an FDA approved drug for the treatment of dry mouth in Sjögren’s syndrome, did show cognitive improvement in the ADAS-cog and word recognition scales in a single-blind-placebo-controlled parallel group study with patients with probable AD (Fisher et al., 1996). Dual selectivity for both the M_1_- and M_2_-receptor on the other hand, as it is the case for RS-86, showed no consistent cognitive improvement in a double-blind, placebo-controlled trial on mild to moderate AD patients (Bruno et al., 1986). Palacios et al. (1986) hypothesized that RS-86’ failure could be due to the concomitant stimulation of the M_1_- and M_2_ receptor, where stimulation of M_2_ might inhibit the effects of the M_1_ receptor. On the other hand, milameline, a partial agonist for all five muscarinic receptor subtypes, demonstrated an effect on the rCBF in the frontal and subcortical regions of AD patients as part of an ‘add-on study’ during a Phase III clinical trial of this drug. AD participants were evaluated with SPECT, using the cerebral blood flow tracer ^99m^Tc-exametazime (^99m^Tc-HMPAO), during the performance of two cognitive tasks. Although a modest increase (of 26%) of rCBF was demonstrated, the authors suggested that there maybe neuropsychopharmacological effects associated with the intake of milameline during the performance of cognitive demanding tasks (Trollor et al., 2006). And finally, in a large-scale clinical trial, xanomeline, a M_1_- and M_4_-receptor agonist and M_5_ receptor antagonist (Grant and El-Fakahany, 2005), was evaluated in a randomized, double-blind, placebo-controlled trial on mild to moderate AD patients. Significant cognitive improvement was hereby shown in the ADAS-cog (drug vs. placebo; *p* ≤ 0.05), and the CIBIC+ scale (drug vs. placebo; *p* ≤ 0.02), demonstrating that a muscarinic receptor agonist can ameliorate cognitive symptoms in AD patients (Bodick et al., 1997).

**FIGURE 3:**
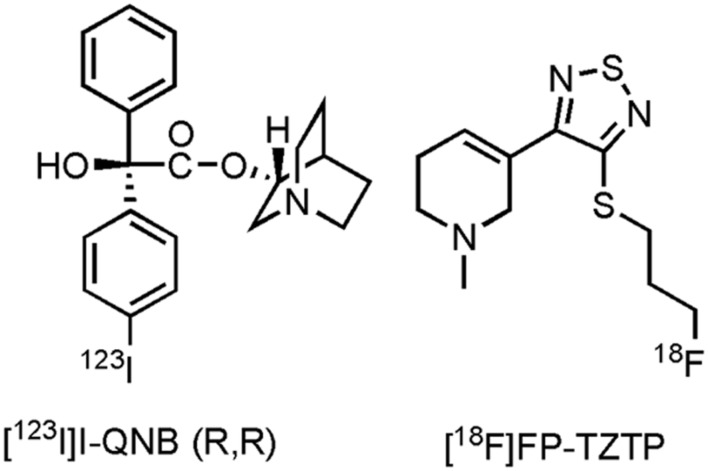
**Structure of muscarinic PET/SPECT tracers**.

Only one imaging agent, with affinity for the M_1_- and M_4_ muscarinic receptor (Piggott et al., 2002), has been clinically evaluated on AD patients thus far, namely [^123^I]I-quinuclidinyl benzilate ((R,R) [^123^I]I-QNB) (**Figure 3**). Eighteen mild to moderate AD patients and their age-matched HCs were evaluated with [^123^I]I-QNB. Significantly reduced uptake (*p* ≤ 0.001) was noted in the frontal rectal gyrus, right parahippocampal gyrus, left hippocampus, and regions of the left temporal lobe, compared to the HCs (Pakrasi et al., 2007). Nevertheless, conflicting results have been reported by other research groups with this tracer (Holman et al., 1985; Weinberger et al., 1991; Wyper et al., 1993; Kemp et al., 2003), although the sample sizes in these other studies were smaller. In another study, [^123^I]I-QNB was used as a biomarker to evaluate the density of the mAChR on 20 patients receiving the AChE inhibitor donepezil (Brown et al., 2003). No distinction could be made between donepezil responders and non-responders, furthermore no positive correlation was found between [^123^I]I-QNB scans and the extent of cognitive improvement on the ADAS-cog scale, apart from the insular cortex, were an inverse correlation was found. Researchers suggest that response to donepezil may thus be greater in patients with clear cholinergic deficits. Several efforts have been made to develop carbon-11 and fluorine-18 labeled tracers for muscarinergic receptors (Farde et al., 1996; Eckelman, 2001; Xie et al., 2004). A clinical trial in the US with3-(3-(3-([^18^F]fluoropropyl)thio)-1,2,5-thiadiazol-4-yl)-1,2,5,6-tetrahydro-1-methylpyridine ([^18^F]FP-TZTP) (Ravasi et al., 2012; **Figure 3**), which binds to the M_2_ receptor, on AD patients has been completed (CTI: NCT00001917), but results are yet to be published. Lastly, recruitment for a study with HCs and AD patients using an M_4_ positive allosteric modulator [^11^C]MK-6884 (structure not yet available), will soon start (CTI: NCT02621606). Future PET- and SPECT compounds hold promise to evaluate inclusion- and outcome criteria with novel drugs targeting the muscarinic receptor, as well as to establish therapeutic windows via dose-occupancy studies.

### Nicotinic ACh Receptor

nAChRs are ionotropic receptors, part of the ligand-gated ion channel superfamily (Cooper and Millar, 1997; Castelan et al., 2008; Criado et al., 2011; Valles and Barrantes, 2012). They consist of a hetero-or homopentameric structure, assembled from 17 possible subunits: α_1-10_, β_1-4_, γ, δ, and ε (Karlin, 2002; Gotti and Clementi, 2004). The main subtypes of the nAChRs in the human CNS are, however, α_7_, α_4_β_2_, and α_3_β_2_, although the latter one is not involved in the pathophysiology of AD (Wevers and Schroder, 1999; Pym et al., 2005). Reduction in nAChRs expression levels of several subtypes has indeed been revealed in regions with dense deposits of Aβ and NFTs (Pimlott et al., 2004; Oddo and Laferla, 2006; Buckingham et al., 2009). The α_7_ nAChR is mainly expressed in the hippocampus, whereas the α_4_β_2_ nAChR is homogenously expressed throughout brain (O’Brien et al., 2007; Valles and Barrantes, 2012). While loss of α_4_β_2_ nAChR can cause memory deficits in AD patients (Paterson and Nordberg, 2000; O’Brien et al., 2007; Kendziorra et al., 2011), a more complex relationship has been noted when evaluating the interaction between α_7_ nAChR and Aβ in AD (Oddo and Laferla, 2006). Aβ can either interact as agonist or as antagonist of α_7_ nAChR, depending on its concentration, where low concentrations may activate and high concentrations may inactivate α_7_ nAChR (Dineley et al., 2002; Puzzo and Arancio, 2013; Sadigh-Eteghad et al., 2014). Observations in an AD transgenic mouse model overexpressing APP, presenilin-1, and tau (3xTg-ADmice) (Oddo et al., 2003; Billings et al., 2005; Kitazawa et al., 2005) were consistent with the aforementioned *in vitro* conclusions for human AD, demonstrating an age dependent reduction of α_7_ nAChR, as higher Aβ may eventually block remaining α_7_ nAChRs (Hernandez et al., 2010). For a more detailed discussion about the many different roles of nAChRs in AD, readers are referred to several reviews on the subject (Oddo and Laferla, 2006; Jurgensen and Ferreira, 2010; Vandenberghe et al., 2010).

Since nicotine can induce the release of presynaptic ACh and is involved in the modulation of many other neurotransmitters, such as GABA, DA, norepinephrine, and serotonin (Levin, 1992), several clinical studies have investigated the effect of nicotine on AD patients (Jones et al., 1992; Wilson et al., 1995; Snaedal et al., 1996; White and Levin, 1999). However, as mentioned in a review by Oddo and Laferla (2006), these trails failed to demonstrate any cognitive improvement in AD patients; only an increase in attention could be determined. Yet, clinical trials with two nAChR agonists did show cognitive improvement in mild to moderate AD patients (Potter et al., 1999; Deardorff et al., 2015). The first of them was encenicline (EVP-6124), a partial agonist of α_7_ nAChR. This drug was well tolerated in Phase I and II trials, showing significant improvement in cognitive and functional domains (Deardorff et al., 2015). A currently ongoing Phase III trial involving mild to moderate AD patients, receiving or having already received AChE inhibitors, to assess the efficacy and tolerability of EVP-6124 (ECT: 2012-003209-92) in a large group of patients was halted due to severe gastrointestinal side-effects (Shugart, 2016). Another nAChR agonist, ABT-418, which has binding affinity for α_4_β_2_, α_2_β_2_, and α_3_β_4_ (Potter et al., 1999), showed some cognitive improvement in the acquisition and retention of verbal information of patients with early AD (Mean MMSE score of 21.4). It is, however, unclear whether this compound will be further pursued in large clinical trials. Two other trials with nAChR agonists were less convincing; efficacy of ispronicline (TC-1734 or AZD-3480) a selective agonist of α_4_β_2_ nAChR and α_2_β_2_ nAChR (Gatto et al., 2004) was investigated in a large Phase IIb dose-finding study on mild to moderate AD patients (MMSE score: 12–26). Despite the fact that ispronicline caused significant improvement on patients with age-associated memory impairment (Dunbar et al., 2007, 2011), no significant improvement could be shown on the ADAS-cog scale in the latest Phase IIb study, although secondary outcome measurements did show some improvement (Frolich et al., 2011). Additional Phase II trials on mild to moderate AD patients were halted, since no superiority over donepezil could be demonstrated. Finally, in a Phase II trial on mild to moderate AD patients (MMSE score of 26–26 and 14–20, respectively), no cognitive improvement could be observed with the α_4_β_2_ nAChR selective agonist varenicline (Kim et al., 2014). Researchers concluded that the dosing regimen was not optimal and overall longer (than 6 weeks) trials may be needed to show cognitive improvement. In the light of these two failed clinical trials with α_4_β_2_ nAChR agonists, another clarification could, however, be that the α_4_β_2_ nAChR subtype may only play a minor role on cognitive processes in AD, and α_7_ nAChR is therefore a more suitable target (Kim et al., 2014).

**FIGURE 4:**
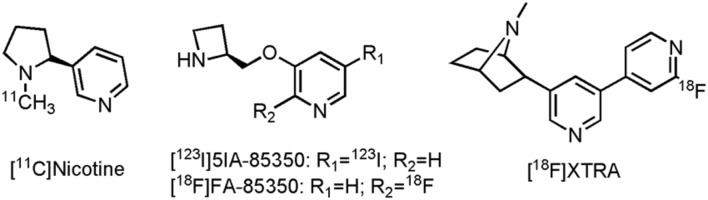
**Structure of nicotinic PET/SPECT tracers**.

While nicotine was not really used as a therapeutic drug, as a carbon-11 labeled PET tracer it successfully showed reduction of nAChR in AD patients reflecting the loss of the nicotinic receptors during disease progression, in comparison with control subjects (Nordberg et al., 1990; Nordberg et al., 1995; Kadir et al., 2006). Additionally, a number of other clinical studies used [^11^C]nicotine PET imaging (**Figure 4**), to assess the efficacy of the AChEIs tacrine and rivastigmine (Nordberg et al., 1992; Nordberg et al., 1997; Nordberg et al., 1998; Kadir et al., 2007). Significant increase in nAChR expression compared to baseline in several cortical areas could be demonstrated, after treatment with both tacrine and rivastigmine. [^11^C]nicotine does, however, show high non-specific binding and rapid brain wash-out, making quantitative PET assessments of nAChR difficult. A few other PET radiotracers are currently under development for imaging of α_7_ and α_4_β_2_ nicotinic receptors (Toyohara et al., 2010; Meyer et al., 2014; Chalon et al., 2015); two structurally related compounds, one SPECT and one PET tracer, with affinity for the α_4_β_2_ nicotinic receptor have already been evaluated on AD patients (O’Brien et al., 2007; Ellis et al., 2008; Sabri et al., 2008). Reduced tracer uptake was noted in AD patients in the frontal lobe, striatum, right medial temporal lobe and the pons after scans with-5-[^123^I] iodo-3-[2(S)-2-azetidinylmethoxy]pyridine ([^123^I]5IA-85350) (**Figure 4**), consistent with known reductions of the α_4_β_2_ nicotinic receptor in AD (O’Brien et al., 2007). Its PET counterpart, 2-[^18^F]fluoro-3-(2(S)-azetidinylmethoxy)pyridine (2-[^18^F]FA-85380) (**Figure 4**), was able to demonstrate significant reduction (up to 75%) of the α_4_β_2_ nicotinic receptor in MCI patients, which later on converted to AD (Sabri et al., 2008). In another study with 2-[^18^F]FA-85380, the possible relationship between Aβ depositions and the reduction of the α_4_β_2_ nicotinic receptor was studied by evaluating early to moderate AD patients (Okada et al., 2013). A negative correlation between the presence of Aβ in the medial frontal cortex and the nucleus basalis magnocellularis, as assessed by [^11^C]Pittsburgh Compound B ([^11^C]PiB, an Aβ tracer), and the binding of 2-[^18^F]FA-85380 to the α_4_β_2_ nicotinic receptor could be established. Both α_4_β_2_ nicotinic receptor tracers suffer, however, from slow kinetics, leading to long scanning times, making routine application difficult (Meyer et al., 2014). Conversely, a novel α_4_β_2_ nAChR tracer, 2-{5-[2-[^18^F]fluoropyridin-4-yl]pyridin-3-yl}-7-methyl-7-azabicyclo[2.2.1]heptane ([^18^F]XTRA) (Meyer et al., 2014) (**Figure 4**), showed much faster pharmacokinetics, which allow scanning within a reasonable time frame. Recruitment for a clinical trial with [^18^F]XTRA on HCs, an AD or a MCI patient is currently ongoing (CTI:NCT01894646).

## Tau Hypothesis

As one of the pathological hallmarks in well over 20 neurodegenerative diseases (Lee et al., 2001), tau (*tubulin associated unit*), gained an increasing interest in the past few years, partly as a result of the large failure rate of clinical trials targeted toward the amyloid hypothesis (Barthel et al., 2015), but also due to recent availability of several tau specific PET ligands (Villemagne et al., 2015). As a member of the microtubule-associated protein (MAP) family, tau is mainly localized in the distal part of the neuronal axons (Binder et al., 1986). Consequently, the primary function of tau is stabilization and support of the microtubules (Weingarten et al., 1975). There are six different isoforms of tau, depending on alternative splicing of exon two, three, and ten of the MAPT. Localized on chromosome 17q32, this gene contains 16 exons (Neve et al., 1986). Exon two and three both encode a 29-aminoacid fragment at the N-terminal part of tau, yielding isoforms with none (0N), one (1N), or two inserts (2N). Exon ten on the other hand, encodes a 31-aminoacid fragment, which results in either three (3R) or four (4R) repeated binding domains at the C-terminus of the protein. The mature human brain contains thus six isoforms of tau: 0N3R, 1N3R, 2N3R, 0N4R, 1N4R, and 2N4R. Under normal conditions (and in AD) there is a 1:1 ratio of the 3R- and 4R isoforms, but this ratio somehow shifts in certain pathological conditions (Hong et al., 1998). The exact physiological role of these various isoforms remains to be elucidated, although 4R isoforms are better at promoting microtubuli assembly, and have greater binding affinity for microtubuli than the 3R isoforms (Goedert and Jakes, 1990; Butner and Kirschner, 1991). Under non-pathologic conditions, tau is a highly soluble protein with a limited secondary structure (Dunker et al., 2008), prone to several post-translational modifications. The most important one is phosphorylation on its serine and threonine residues, which modulates microtubule binding (Martin et al., 2011). Then again, in pathological conditions, such as AD, tau will become hyperphosphorylated, detaches from the microtubules and will self-aggregate into insoluble PHFs and NFTs, compromising neuronal cell function (Iqbal and Grundke-Iqbal, 2008). Different shapes and sizes of the aggregates can be found under diverse cognitive conditions, related to the presence of various isoforms, and post-translational modifications (Ballatore et al., 2007). In AD, the spreading of the tau pathology, which is thought to proceed in a prion-like manner (de Calignon et al., 2012), has been well-documented in the different Braak stages (Braak and Braak, 1991). Furthermore, several studies confirmed that this characteristic pattern of aggregated tau spread closely correlates to the clinical symptoms of AD, as measured by the MMSE (Bancher et al., 1993, 1996; Duyckaerts et al., 1997; Grober et al., 1999). This makes tau an interesting target for drug development. Due to the complexity of aggregated tau as a drug target, several tau-associated approaches have been investigated.

Glycogen synthase kinase 3β, being the dominant isoform of three GSK-3 variants (Jaworski et al., 2011), is the main kinase responsible for the hyperphosphorylation of tau, and hence an important potential target for disease-modification (Maqbool et al., 2016). Diverse GSK-3 inhibitors were reported the last few years by many research groups (Noble et al., 2011; Berg et al., 2012; Maqbool et al., 2016). Lithium and valproate were the first compounds to be clinically evaluated, but due to inconsistent and overall disappointing results, they were largely discontinued (Noble et al., 2005; Hampel et al., 2009; Tariot and Aisen, 2009; Tariot et al., 2011). Several other GSK-3 inhibitors have, however, been pursued (Maqbool et al., 2016), two of which, tideglusib (NP0311212) and AZD1080, entered clinical trials (King et al., 2014; Lovestone et al., 2015). Development of AZD1080 was, however, halted in Phase I due to nephrotoxicity problems (Eldar-Finkelman and Martinez, 2011) and no clinical benefit was seen with tidelusib on patients with mild to moderate AD in Phase II clinical trials. Dose finding studies and longer trials are now required with the latter drug to examine its possible long term benefit (Lovestone et al., 2015). Another tau-associated approach is the inhibition of tau-aggregation (Bulic et al., 2009, 2010). The first of this class to be pushed in Phase II clinical trials, methylthioninium chloride (methylene blue, MTC), a phenothiazine derivative, was able to stabilize disease progression over a period of 50 weeks in mild and moderate AD patients (Wischik et al., 2015). The brain bioavailability of this charged drug remains, however, to be elucidated. The pro-drug of MTC, leuco-methylthioninium (TRx0237 or LMTX), with a superior pharmacological profile (Wischik et al., 2014) will now be evaluated in three parallel Phase III trials on mild to moderate AD patients and patients with FTD (CTI: NCT01689246, NCT01689233, and NCT01626378). And finally, an increasing interest toward tau immunotherapy has been noted, as means of removing tau aggregation by the patients’ own immune system (Asuni et al., 2007). Two drugs of this kind, ACI-35 (ECT: 2015-000630-30) and AADvac1 (CTI: NCT02031198) are currently being evaluated in Phase I and II trials.

**FIGURE 5:**
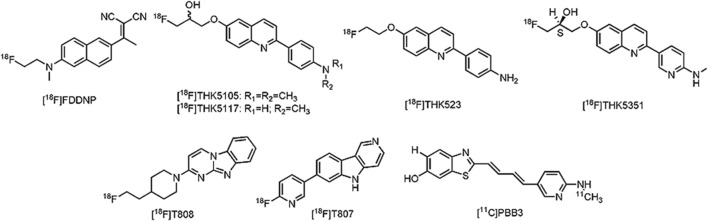
**Structure of tau PET tracers**.

Despite the historical importance of tau as a pathological hallmark in AD (Graeber and Mehraein, 1999), only recently tau specific PET ligands have been developed. One of major issues during tau PET development is the lack of a representative tau-animal model, which may be explained by (ultra)structural differences between murine and humane tau (Duyckaerts et al., 2008). More than a few tracers are, however, currently being clinically evaluated. The first tau PET ligand to be reported was 2-(1-{6-[(2-[^18^F]fluoroethyl)(methyl)amino]-2-naphthyl}ethylidene)malononitrile ([^18^F]FDDNP) (**Figure 5**), although not specific for tau as such, high binding affinity was reported in several neurodegenerative diseases (Bresjanac et al., 2003; Small et al., 2006; Kepe et al., 2010; Nelson et al., 2011; Kepe et al., 2013; Small et al., 2013). Limited dynamic range of signal and relatively low affinity for tau, led to the development of novel tau directed ligands with similar structural moieties. Yet, the first real approach toward tau specific ligands was achieved by researchers of the Tohoku University in Japan with the development of 4-{6-[2-[^18^F]fluoroethoxy]quinolin-2-yl}aniline ([^18^F]THK523) (Fodero-Tavoletti et al., 2011) (**Figure 5**). While [^18^F]THK523 was able to visualize the known pattern of tau distribution in AD patients, high white matter binding and unfavorable pharmacokinetics (Villemagne et al., 2014) led to the development of three novel 2-arylquinoline derivatives: 1-({2-[4-(dimethylamino)phenyl]quinolin-6-yl}oxy)-3-[^18^F]fluoropropan-2-ol ([^18^F]THK5105) (Okamura et al., 2014a), 1-[^18^F]fluoro-3-({2-[4-(methylamino)phenyl]quinolin-6-yl}oxy)propan-2-ol ([^18^F]THK5117) (Ishiki et al., 2015) and the optically pure (2S)-1-[^18^F]fluoro-3-({2-[4-(methylamino)phenyl]quinolin-6-yl}oxy)propan-2-ol ([^18^F]THK5351) (Harada et al., 2016) (**Figure 5**). All three compounds showed high binding in AD patients, with radiotracer retention in sites known for their tau deposition. Of these three compounds, [^18^F]THK5351 showed superior pharmacokinetics, highest signal-to-noise ratio and the lowest white matter binding (Okamura et al., 2014b; Harada et al., 2016). Further clinical trials in Japan with [^18^F]THK5351 are underway (UMIN-CTR: UMIN000013929 and UMIN000018496). Often considered by many research groups as the current benchmark in tau PET development, 11-{4-[2-[^18^F]fluoroethyl]piperidin-1-yl}-1,8,10-triazatricyclo[7.4.0.0^2,7^]trideca-2(7),3,5,8,10,12-hexaene ([^18^F]T808 or [^18^F]AV680), and 2-[^18^F]fluoro-5-{5H-pyrido[4,3-b]indol-7-yl}pyridine ([^18^F]T807 or [^18^F]AV1451) (**Figure 5**) have high affinity and selectivity for tau over Aβ (Zhang et al., 2012; Xia et al., 2013; Shah and Catafau, 2014). A small first-in-man study with [^18^F]T808 in eight AD patients (mean MMSE of 18) and their three age matched HCs, showed a rapid brain uptake and washout in HCs, and a tau pattern consistent with the Braak stages in the AD group (Chien et al., 2013). Interestingly, one of the AD patients who died a few weeks after his PET scan with [^18^F]T808, showed close correlation with his histopathological staining (Dani et al., 2015). Nonetheless, substantial bone uptake was observed with this compound (Villemagne et al., 2015), which led to the development of [^18^F]T807. In comparison to [^18^F]T808, [^18^F]T807 has slower kinetics and a relatively lower affinity for tau, but [^18^F]T807 does not show defluorination (Xia et al., 2013). Similar clinical findings as with [^18^F]T808 were demonstrated with [^18^F]T807 in a small first-in-man study on three HCs, in one patient with MCI (MMSE score of 26) and one severe AD patient (MMSE score of 7) in comparison with three HCs. Remarkably, the tracer retention was significantly lower in the patient with MCI, as compared to the patient with severe AD (Chien et al., 2012). A series of large clinical trials (ClinicalTrial.gov and EU Clinical Trials Register: search term: ‘T807’ OR ‘AV1451’ AND ‘PET’) is underway with [^18^F]T807 to evaluate its applicability not only in AD, but also in several other tauopathies. Being thus far the only compound to be able to visualize tau (and possibly different isoforms) in AD, but also in PSP and CBD (Maruyama et al., 2013), 2-((1E, 3E)-4-(6-([^11^C]methylamino)pyridin-3-yl)buta-1,3-dienyl)benzo[d]thiazol-6-ol ([^11^C]PBB3) received a lot of interest (**Figure 5**). Clinical studies on AD patients and a CBD patient, as compared to HCs, showed increased tracer uptake, consistent with the Braak stages (for the AD case), and higher retention in the basal ganglia (for the CBD case). Stability issues and a challenging radiosynthesis might, however, limit its commercial use. Several other tau directed PET ligands from Roche, such as [^11^C]RO6931643, [^11^C]RO6924963, and [^18^F]RO6958948 (structures not available) have been evaluated in a Phase I clinical trial, but data are yet to be published (CTI: NCT02187627) (Dani et al., 2015). Other clinical studies with [^18^F]MK-6240, [^18^F]MNI-798, and [^18^F]MNI-815 (structures not available) on AD cases are currently recruiting patients (CTI: NCT02562989, NCT02640092, and NCT02531360). For a more detailed discussion about the current state of tau PET development, readers are referred to some excellent reviews on the subject (Okamura et al., 2014b; Zimmer et al., 2014; Dani et al., 2015). An important question that remains to be elucidated is for which isoforms these tau PET tracers have affinity; a question which may have major implications on the differential diagnosis of closely related neurodegenerative tauopathies. Nevertheless, the substantial progress that has been made in this field will make it possible to allow *in vivo* detection of tau in AD and thus the reassessment of inclusion- and outcome criteria of clinical trials aiming to intervene at tau-aggregates.

## Amyloid Hypothesis

For many decades, the amyloid hypothesis has been the main pathological model of AD (Hardy and Higgins, 1992; Korczyn, 2008), accepted by most researchers, and only recently contested (Hardy, 2006). It postulates that extensive deposits of amyloid in the human brain are the central lesions in the development of AD, responsible for a neurotoxic cascade of events, which ultimately leads to dementia (Barage and Sonawane, 2015). Aβ peptides, the main component of SP (Gomez-Isla et al., 1997), are 39–43 amino acid residues, formed during the sequential cleavage of the transmembrane APP by β-secretase 1 (also called BACE1) (Haass, 2004), followed by the action of the γ-secretase (Selkoe, 2001). Under ‘normal’ conditions APP is cleaved in a non-amyloidogenic pathway by the action of initially α-secretase, forming α-sAPP, which may have a neuroprotective function (Pagani and Eckert, 2011). α-sAPP is then further cleaved by γ-secretase to eventually produce P3. The function of APP itself is unknown, although a possible role in the Cu-homeostasis has been proposed (Barnham et al., 2004). There are two main isoforms of Aβ: Aβ_40_ and Aβ_42_, the latter one being more prone to aggregation and regarded as the main neurotoxic species (Ballard et al., 2011). Once formed, Aβ species will undergo several characteristic changes, from small oligomers into larger fibrils, which eventually form diffuse and later neuritic plaques. These plaques frequently trigger astrocytosis, activation of microglial cells, cytokine release, and a multi-protein neuroinflammatory response (Barage and Sonawane, 2015). Just like the NFTs, amyloid depositions follow a specific pattern, as recorded by Braak and Braak (1991). In contrast to the NFTs, however, there is a poor correlation between the extent of these plaques and the degree of cognitive impairment (Nelson et al., 2012), furthermore non-demented individuals can heave substantial loads of Aβ deposition without revealing any clinical symptoms (Villemagne et al., 2008). Recently, several studies pointed toward Aβ oligomers, and not amyloid plaques, as the main toxic species in AD (Haass and Selkoe, 2007; Minati et al., 2009). For a more extensive discussion about the neuropathological role Aβ plays in AD, readers are referred to other reviews (Haass and Selkoe, 2007; Hardy, 2009; Barage and Sonawane, 2015).

Huge efforts have been undertaken to develop disease altering drugs that target the amyloid deposition, but unfortunately many failed during clinical trials (Barthel et al., 2015). Some of the most recent *ongoing* trials targeting the amyloid deposits are summarized in **Table 1**. Various therapeutic approaches are to be considered when targeting Aβ (Barage and Sonawane, 2015). We will discuss here the most important tactics, together with some of their constraints, as it is imperative to know why so many trials fail in this area. One of many methods applied, is the reduction of Aβ production through inhibition of β-and/or γ-secretase or activation of α-secretase (Cummings, 2008). Indeed, the therapeutic potential of BACE1 inhibitors has been demonstrated in BACE1 knockout mice, which produced significantly (15-fold) less Aβ (Luo et al., 2001; Roberds et al., 2001). Nonetheless, inhibition of BACE1 causes several problems, since BACE1 has been shown to have many physiological roles, which might lead to toxicity problems when using BACE1 inhibitors. Furthermore, BACE1 inhibitors need to be quite bulky, due to the relatively large active site and this can cause BBB passage issues (Ghezzi et al., 2013). Inhibition of the multimeric γ-secretase complex encounters similar problems as the use of BACE1 inhibitors, as γ-secretase has many other physiological roles as well, especially cleavage of the Notch receptor, necessary for growth and development (Yiannopoulou and Papageorgiou, 2013). Brain penetration seems to be an issue as well in this area (Imbimbo and Giardina, 2011). Increasing the α-secretase activity, and thus promoting the non-amyloidogenic pathway, is another way to reduce the Aβ load. Less is however known about the possible physiological consequences of such an upregulation (Barage and Sonawane, 2015). Another way of interfering with the Aβ load is by modulation of Aβ aggregation, as increasing evidence suggests that soluble oligomers, which act as intermediates for the formation of aggregates, are the most toxic species in AD disease (Dahlgren et al., 2002; Hoshi et al., 2003; Kayed et al., 2003). Yet, the most promising small molecules ultimately failed due to their (toxic) pharmacological profile (Santa-Maria et al., 2007; Rishton, 2008; Yiannopoulou and Papageorgiou, 2013). Several studies also showed the relationship of APP and Aβ with mitochondrial dysfunction in AD (Anandatheerthavarada et al., 2003; Lustbader et al., 2004; Caspersen et al., 2005); interaction of both proteins with mitochondrial matrix proteins, such as Aβ-binding alcohol dehydrogenase and adenosine triphosphate synthase subunit alpha, could directly lead to mitochondrial toxicity, and thus oxidative stress (Devi et al., 2006; Reddy and Beal, 2008). Numerous antioxidant agents have been described and evaluated in clinical trials with MCI- and AD patients, and several studies are still ongoing (Mecocci and Polidori, 2012; Polidori and Nelles, 2014). Conflicting results were, however, reported and overall small cognitive benefit was seen during these trials. Long-term trails are now warranted in order to establish clinical benefits in AD. Then again, several promising antioxidative compounds are currently being investigated (Qosa et al., 2015; Rigacci, 2015). Still, the primary action in targeting amyloid came from monoclonal antibodies. First discovered by Schenk et al. (1999) to be very effective in reducing the Aβ load in mice, the mechanism of action of amyloid immunotherapy remains, however, to be fully elucidated (Yiannopoulou and Papageorgiou, 2013). Nevertheless, only a small fraction (0.1% of the injected dose) of antibodies seems to be able to pass the BBB in humans (Banks et al., 2002). A higher fraction of antibodies in the brain may thus be needed to be therapeutically effective. This hurdle was the topic of two recently reported reviews (Lemere, 2013; Spencer and Masliah, 2014). Despite the low BBB’s passage, one of the major concerns with immunotherapy is the development of serious side effects, for instance encephalitis (active immunization), microhemorrhages, or vasogenic edemas (passive immunization) (Orgogozo et al., 2003; Panza et al., 2012). Another important factor to consider is the time of intervention in the AD state; immunotherapy is probably most efficacious in early disease states, when there is more function to preserve (Barthel et al., 2015). Efficient biomarkers, that can predict the conversion from MCI to AD, are therefore of utter importance. Using longitudinal PET biomarkers to assess and follow up the amyloid burden in clinical trials would indeed allow a more confident formulation of inclusion, but also outcome criteria (Barthel et al., 2015). Several amyloid PET tracers are currently being used for these purposes (see **Table 1**).

**Table 1:** Ongoing clinical trials with drugs targeting the amyloid hypothesis (Han and Mook-Jung, 2014; Wischik et al., 2014; Apter et al., 2015).

Drug	Approach	Trial phase	CTI	PET biomarker*	EudraCT number
MK-8931	BACE1 inhibitor	3	NCT01953601	[^18^F]flutemetamol	2012-005542-38
		2/3	NCT01739348	[^18^F]flutemetamol	2011-003151-20
AZD3293	BACE1 inhibitor	2/3	NCT02245737	[^18^F]AV-45 / [^18^F]FDG	2014-002601-38
PF-03084014	γ-secretase inhibitor	2	NCT01981551	Not specified	/
NIC5-15	γ-secretase inhibitor	2	NCT01928420	Not specified	/
Bryostatin-1	α-secretase enhancer	2	NCT00606164	Not specified	/
		2	NCT02431468	Not specified	/
Solanezumab	Passive immunization	2/3	NCT01760005	[^11^C]PiB / [^18^F]FDG	2013-000307-17
		3	NCT01900665	[^18^F]AV-45	2013-001119-54
Gantenerumab	Passive immunization	3	NCT02051608	[^18^F]AV-45	2013-003390-95
		3	NCT01224106	Not specified	2010-019895-66
		2/3	NCT01760005	[^11^C]PiB / [^18^F]FDG	2013-000307-17
Crenezumab	Passive immunization	1	NCT02353598	[^18^F]AV-45	/
		2	NCT01998841	Not specified	/
		2	NCT01723826^C^	Not specified	2012-003242-33
BAN2401	Passive immunization	2	NCT01767311	Not specified	2012-002843-11
Gammagard	Passive immunization	2/3	NCT01561053	[^18^F]FDG	/
Aducanumab	Passive immunization	3	NCT02484547	Not specified	2015-000967-15
		3	NCT02477800	Not specified	2015-000966-72
		1	NCT02434718	Not specified	/

*C = Completed; PET biomarkers are either used as inclusion criteria and/or as outcome measure.

**FIGURE 6:**
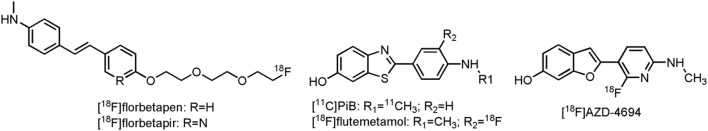
**Structure of Aβ PET tracers**.

Although not FDA-approved, 2-{4-[[^11^C]methylamino]phenyl}-1,3-benzothiazol-6-ol ([^11^C]PiB) (**Figure 6**) has been used for many years as benchmark compound for *in vivo* imaging of the amyloid load in AD patients (Benadiba et al., 2012). Results of those trials have shown that clinically diagnosed AD cases have positive amyloid scans (Kemppainen et al., 2006; Jack et al., 2008, 2009; Lowe et al., 2009), and the ones that did not have positive scans, were most likely to be misdiagnosed (Rabinovici et al., 2007; Rabinovici et al., 2008). Furthermore, increased [^11^C]PiB binding is able to predict the conversion of MCI to AD (Okello et al., 2009). [^11^C]PiB has also been proven useful in the differential diagnosis of FTD and AD, as FTD patients typically have a normal [^11^C]PiB uptake (Rowe et al., 2007; Engler et al., 2008). Interestingly, a close correlation has been noted with CSF Aβ levels (Tolboom et al., 2009; Weigand et al., 2011), firmly establishing [^11^C]PiB as an Aβ biomarker. There are, however, a few limitations with [^11^C]PiB as an amyloid biomarker; [^11^C]PiB presumably binds to diffuse plaques and not to the more cognitive correlated neuritic plaques (Jack et al., 2013). Moreover, commercial use is excluded, due to the short half-life of carbon-11. Several attempts were thus undertaken to develop ^18^F-labeled analogs (Koo and Byun, 2013). Three of them: 4-[(E)-2-[6-(2-{2-[2-[^18^F]fluoroethoxy]ethoxy}ethoxy)pyridin-3-yl]ethenyl]-*N*-methylaniline ([^18^F]florbetapir or [^18^F]AV-45), 2-[3-[^18^F]fluoro-4-(methylamino)phenyl]-1,3-benzothiazol-6-ol ([^18^F]flutemetamol, [^18^F]GE-067 or [^18^F]AV-1) and 4-[(E)-2-[4-(2-{2-[2-[^18^F]fluoroethoxy]ethoxy}ethoxy)phenyl]ethenyl]-*N*-methylaniline([^18^F]florbetaben or [^18^F]BAY 97-9172) (**Figure 6**) have already been approved by the FDA and the EMA for their binding to neuritic plaques. Another one, 2-[2-[^18^F]fluoro-6-(methylamino)pyridin-3-yl]-1-benzofuran-6-ol ([^18^F]AZD-4694 or [^18^F]NAV4694) (**Figure 6**), is currently awaiting FDA-approval (Jack et al., 2013). Although all of the current ^18^F-labeled compounds show significant increased uptake in AD patients as compared to HCs in clinical trials (Rowe et al., 2008, 2013; Barthel et al., 2011; Villemagne et al., 2011), they suffer from high non-specific white matter binding, as compared to [^11^C]PiB (Benadiba et al., 2012; Rowe and Villemagne, 2013; Vandenberghe et al., 2013). Only [^18^F]AZD-4694 has a white matter uptake similar to [^11^C]PiB (Rowe et al., 2013). While a negative amyloid PET scan will exclude AD, a positive scan, on its own, is insuffient for the diagnosis of AD, as has been shown with [^18^F]florbetapir in clinic (Yang et al., 2012). Furthermore, limited reimbursement of these recently approved compounds limits their use in clinical practice (Barthel et al., 2015). The use of amyloid PET may, however, reveal true AD cases. Moreover, the amyloid tracers are able to predict the conversion from MCI to AD, and this can considerably influence decision making in AD related clinical trials (Rowe and Villemagne, 2013).

## Gamma-Aminobutyric Acid Receptors

The inhibitory GABA system in the CNS consists of three GABA receptor systems: GABA_A_, GABA_B_ and GABA_C_ (Chebib and Johnston, 1999). Since GABA_B_- and GABA_C_ receptors have not been clinically evaluated in AD yet, focus will be toward the GABA_A_ receptor. The GABA_A_ receptor is a pentameric ligand gated ion channel, composed of a wide array of (possible) subunits: α_1-6_, β_1-3_, γ_1-3_, δ, ε, τ, π, and ρ_1-3_ (Mehta and Ticku, 1999). In order to be functional, the receptor seems to require the presence of at least one α- and one β-subunit. The most common composition is a pentamer composed of two α-, two β-, and one γ-subunit (Connolly et al., 1996). The GABA system plays an important role in AD, as it is one of the main culprits for the BPSD. Contributing to these BPSD, the GABA system is also known to modulate other neurotransmitters, such as serotonin, DA and ACh (Decker and McGaugh, 1991; Zorumski and Isenberg, 1991; Keverne, 1999). It has been a long standing, although contested (Lanctot et al., 2004), view that the GABA system undergoes little change during AD progression, due to dynamic plasticity of the system (Rissman et al., 2007). Recent findings suggest, however, otherwise and point to a severely altered GABAergic signaling in AD, with possible modulation of tau hyperphosphorylation (Lanctot et al., 2004; Limon et al., 2012; Nykanen et al., 2012). A more detailed discussion of the GABAsystem and its putative role in AD can be found in some extensive reviews (Marczynski, 1998; Lanctot et al., 2004; Rissman et al., 2007).

Benzodiazepines, which are allosteric modulators of the GABA_A_ receptor (Hevers and Luddens, 1998), have long been used for the symptomatic treatment of anxiety and agitation in AD (Kirven and Montero, 1973; Covington, 1975; Sunderland et al., 1989; Zec and Burkett, 2008), nevertheless there is a need for randomized controlled trials to evaluate the true efficacy of these drugs in AD (Defrancesco et al., 2015). Caution is also to be advised with BZDs, as there are reports of rapid cognitive and functional decline in AD patients when taking these drugs for an extensive period of time (Zec and Burkett, 2008).

**FIGURE 7:**
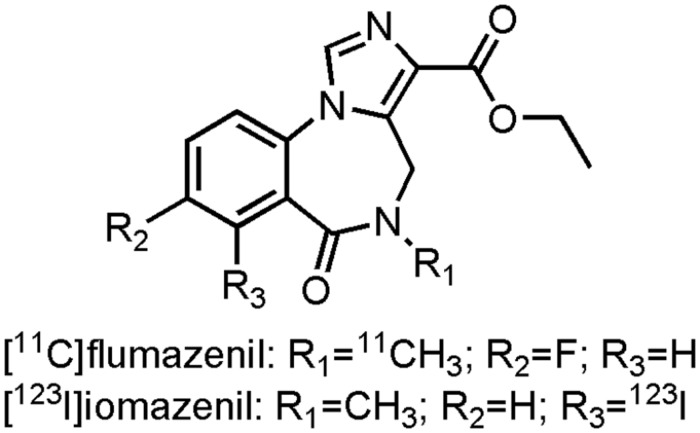
**Structure of GABAergic PET/SPECT tracers**.

There have been numerous endeavors to developed radiotracers for *in vivo* imaging of the GABA_A_ receptors (Katsifis and Kassiou, 2004; Andersson and Halldin, 2013). Ethyl 12-fluoro-8-[^11^C]methyl-9-oxo-2,4,8-triazatricyclo[8.4.0.02^2,6^]tetradeca-1,3,5,10,12-pentaene-5-carboxylate ([^11^C]flumazenil) (**Figure 7**), a GABA_A_antagonist with affinity for the α_1-3_ and α_5_-subunit, is the most promising tracer thus far. Several clinical studies have been performed with [^11^C]flumazenil (Savic et al., 1988; Heiss et al., 2004; Frankle et al., 2009, 2012; Andersson and Halldin, 2013), one of which was carried out on early AD patients (Mean MMSE: 21.2) to evaluate the GABA_A_ receptor density. Researches demonstrated a marked decrease in [^11^C]flumazenil binding, which correlated well with neuronal loss as evaluated by histopathological findings (Brun and Englund, 1981; Andersson and Halldin, 2013). The SPECT analog ethyl 11-[^123^I]iodo-8-methyl-9-oxo-2,4,8-triazatricyclo[8.4.0.0^2,6^]tetradeca-1,3,5,10,12-pentaene-5-carboxylate ([^123^I]iomazenil) (**Figure 7**), showed significantly reduced uptake in the temporal, parietal end occipital cortex of moderate to severe AD patients (Soricelli et al., 1996; Fukuchi et al., 1997). In contrast to [^11^C]flumazenil though, [^123^I]iomazenil was not able to show significant changes in early AD patients (Pappata et al., 2010). Interestingly, in a direct PET-SPECT comparison study on healthy volunteers between [^11^C]flumazenil and [^123^I]iomazenil, the ^123^I-labeled variant came out as the better candidate, due to a better fit in compartmental modeling with SPECT (Bremner et al., 1999). These radiopharmaceuticals not only hold promise to be used as inclusion- and outcome criteria for drugs combatting BPSD symptoms in AD, but they could also be used in dose-occupancy studies to assess the (sometimes small) therapeutic window of BZDs.

## Serotonergic System

Serotonin (5-HT), is a neurotransmitter that plays a complex role in the modulation of several psychological, emotional, and cognitive processes. Moreover, 5-HT affects long-term and short-term memory and cognitive function through the regulating of many other neurotransmitters, such as ACh, DA, GABA, and glutamate (Rodríguez et al., 2012). The principal 5-HT-source in the human brain comes from neurons in the raphe nuclei, with various projections throughout the CNS (Vertes, 1991; Vertes et al., 1999). There are seven main 5-HTRs, which can be divided into two major classes: the G-protein coupled receptors (5-HTR_1,2,4-7_) and the ligand-gated cation channels (5-HTR_3_), many of which have also several subcategories (Hoyer et al., 2002). For the ‘normal’ physiological and pharmacological role of these receptors, readers are referred to several reviews on the topic (Barnes and Sharp, 1999; Hoyer et al., 2002; Niesler et al., 2008). An overall reduction of the serotonergic system in AD pathology, likely reflecting the loss of serotonergic projections from the raphe nuclei, has been demonstrated (Bowen et al., 1983; Chen et al., 1996, 2000). Interestingly, loss of function seems more extensive in early onset AD than in later-onset AD, which may be due to compensating systems (Arai et al., 1992; Halliday et al., 1992). More specifically, marked reduction of the 5-HTR_1A_, which is expressed in brain areas known for their role in memory and learning, has been noted in the hippocampus and the frontal cortex during AD progression (Lai et al., 2003). This may, however, reflect a compensatory mechanism for reduction of cholinergic receptors in the AD brain, since inhibition of the 5-HTR_1A_ has been implicated in the release of ACh (Millan et al., 2004; Kehr et al., 2010; Rodríguez et al., 2012). Another receptor that is affected during AD progression is 5-HTR_2A_, with reductions being noted in the frontal, temporal, parietal and enthorinal cortex and the hippocampus (Crow et al., 1984; Procter et al., 1988; Dewar et al., 1990). In a review by Rodríguez et al. (2012) it was suggested that a decrease in 5-HTR_2A_ may effect cognitive functions in AD patients. A positive correlation between cognitive decline and 5-HTR_2A_ related decrease in the frontal cortex has indeed been noted (Lai et al., 2005). Moreover, it was implied that decrease in 5-HTR_2A_ density may be due to pathological accumulation of Aβ (Christensen et al., 2008; Holm et al., 2010). Stimulation of the 5-HTR_4_ may lead to an increase of the non-amyloidogenic pathway *in vitro* (Consolo et al., 1994; Robert and Benoit, 2008), indication for an important role in APP metabolism. Other 5-HTRs with marked reduction in AD are 5-HTR_1B_, 5-HTR_1D_, and 5-HTR_6_ (Garcia-Alloza et al., 2004; Lorke et al., 2006). Additionally, reduction of the former two correlates well with the cognitive decline in AD (Garcia-Alloza et al., 2004). Significant decrease (up to 25%) in binding sites of the 5-HTT during AD progression is also to be noted (Bowen et al., 1983; Ouchi et al., 2009). As part of the monoamine transporter family, the SERT is responsible for removal of serotonin from the synaptic cleft. There seems, however, no correlation between the reduced density of this transporter and BPSD symptoms, as seen in AD (Tsang et al., 2010).

Most drugs targeting the serotonergic system are used as adjuvant therapy, combatting BPSD symptoms by inhibition of SERT and/or the norepinephrine transporter. Recent meta-analyses have proven their efficacy in treating these behavioral symptoms in AD (Ballard and Corbett, 2010; Henry et al., 2011). Some serotonin reuptake inhibitors have, however, also been evaluated for their possible cognitive enhancement in AD patients (See **Table 2**). Likewise, increasing interest has been noted for 5-HTR drugs that are able to improve cognition and/or memory in AD. 5-HT_1_-, 5-HT_4_-, and 5-HT_6_ receptors are hereby of particular interest, due to their important role in learning and memory processes (Geldenhuys and Van der Schyf, 2011), effects which are most likely due to their modulation on glutamatergic and cholinergic transmission, or, in the case of 5-HT-4, due to an enhanced release of ACh upon stimulation of this receptor (Rodríguez et al., 2012). An overview of trials that have looked into the clinical benefit of serotonergic drugs on cognitive impairment in AD patients is given in **Table 2**.

**Table 2:** Enhancement of cognitive functions in AD by drugs that modulate serotonergic neurotransmission (Geldenhuys and Van der Schyf, 2011; Ramirez et al., 2014).

Drug	Mechanism	Trial (Phase)	Outcome	Reference/ongoing trail
Lecozotan (SRR-333)	5-HTR_1A_ antagonist	2	Unsuccessful due to adverse effects	Sabbagh, 2009
Xailiproden (SRR57746A)	5-HTR_1A_ antagonist	3	Unsuccessful to demonstrate efficacy	Sabbagh, 2009
PRX-03140	5-HTR_4_ agonist	2	Improvement on ADAS-cog scale	Sabbagh, 2009
SB-742457	5-HTR_6_ antagonist	2	Improvement on CIBIC+ score and ADAS-cog scale	Maher-Edwards et al., 2010
Lu-AE-58054 (SGS-518)	5-HTR_6_ antagonist	2	Improvement on ADAS-cog scale and ADL	Rodríguez et al., 2012
		3	Ongoing	NCT02079246
		3	Ongoing	NCT02006654
		3	Ongoing	NCT02006641
		3	Ongoing	NCT01955161
PF-05212377 (SAM-760)	5-HTR_6_ antagonist	2	Ongoing	Rodríguez et al., 2012/NCT01712074
SUVN-502	5-HTR_6_ antagonist	2	Ongoing	Geldenhuys and Van der Schyf, 2011/NCT02580305
Citolapram	SSRI	4 weeks	Improvement on ADL	Nyth and Gottfries, 1990
Fluoxetine	SSRI	8 weeks	Improvement on MMSE	Mowla et al., 2007
Sertraline	SSRI	12 weeks	Improvement on ADL	Lyketsos et al., 2003

*ADAS-cog, Alzheimer’s Disease Assessment Scale-cognitive subscale; CIBIC+, Clinician’s Interview-Based Impression of Change; ADL, Activity of Daily Living; MMSE, Mini-Mental State Examination; SSRI, Selective Serotonin Reuptake Inhibitor.*

**FIGURE 8:**
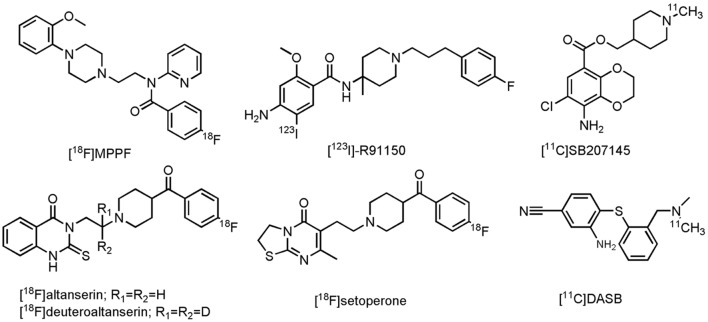
**Structure of serotonergic PET/SPECT tracers**.

Although much progress has been made in the development of PET- and SPECT radioligands for visualization of the serotonergic system (Paterson et al., 2013), only a few radiolabeled compounds have been evaluated on AD patients thus far. In the group of the 5-HT_1A_R, only one PET radioligand, 4-[^18^F]fluoro-*N*-{2-[4-(2-methoxyphenyl)-1-piperazinyl]ethyl}-*N*-(2-pyridinyl)benzamide ([^18^F]MPPF, **Figure 8**), a reversible, competitive 5-HT_1A_R antagonist, was investigated in patients with MCI and AD (Kepe et al., 2006; Truchot et al., 2008). Decrease of [^18^F]MPPF binding was noticed in the hippocampus and raphe nuclei of AD patients (as compared to HCs). Furthermore, loss of receptor density in the hippocampus was strongly correlated to a decline in the MMSE score. In patients with MCI, only a small loss of 5-HT_1A_R density was noticed, correlated to only small cognitive decline (Kepe et al., 2006). [^18^F]MPPF is one of many fluoro-analogs of [^11^C]WAY100635, the latter one being excessively studied in humans. Yet, no studies on AD patients were performed with [^11^C]WAY100635, mainly due its rapid metabolism, making kinetic modeling difficult (Paterson et al., 2013). [^18^F]MPPF does not suffer from these limitations, but is on the other hand a substrate of the P-gp, which could limit its further use in clinic (Kumar and Mann, 2014). Imaging of the 5-HT_2_R in AD patients was done by one SPECT- and three PET radiolabeled 5-HT_2_R antagonists, namely 4-amino-*N*-{1-[3-(4-fluorophenyl)propyl]-4-methylpiperidin-4-yl}-5-[^123^I]iodo-2-methoxybenzamide ([^123^I]-R91150), 6-(2-{4-[4-[^18^F]fluorobenzoyl]piperidin-1-yl}ethyl)-7-methyl-2H,3H,5H-[1,3]thiazolo[3,2-a]pyrimidin-5-one ([^18^F]setoperone), 3-(2-{4-[4-[^18^F]fluorobenzoyl]piperidin-1-yl}ethyl)-2-sulfanylidene-1,2,3,4-tetrahydroquinazolin-4-one ([^18^F]altanserin) and 3-(2-{4-[4-[^18^F]fluorobenzoyl]piperidin-1-yl}(2,2-^2^H2)ethyl)-2-sulfanylidene-1,2,3,4-tetrahydroquinazolin-4-one ([^18^F]deuteroaltanserin) (**Figure 8**). In agreement with previous postmortem studies, an overall significant reduction in the cerebral cortex was noted in mild to severe AD patients, compared to their age-matched controls (Blin et al., 1993; Versijpt et al., 2003; Santhosh et al., 2009; Marner et al., 2012). In the 5-HT_4_R class though, one PET ligand was evaluated on AD patients: [1-[^11^C]methylpiperidin-4-yl]methyl 8-amino-7-chloro-2,3-dihydro-1,4-benzodioxine-5-carboxylate ([^11^C]SB207145), a 5-HT_4_R antagonist (**Figure 8**). This radioligand did not display significant differences between mild AD cases and their HCs, although a positive correlation was found with the Aβ density (as measured by [^11^C]PiB). Moreover, a negative correlation was noticed between [^11^C]SB207145’s binding potential and the MMSE score. Authors suggested that upregulation of 5-HT_4_R may take place at a preclinical stage of AD (this in contrast to the other 5-HTRs) and that this may continue through the later AD stages (Madsen et al., 2011). Finally, (3-amino-4-(2-dimethylamino-methyl-phenylsulfanyl)-benzonitrile) ([^11^C]DASB, **Figure 8**), a SERT tracer, displayed a more outspoken decrease (25%) of binding in the subcortical serotonergic projection region in depressed, as compared to non-depressed AD patients (mean MMSE score of 18) (Ouchi et al., 2009). Yet, in another clinical study on patients with mild AD (not corrected for depression) no such reduction was found (Marner et al., 2012). Authors of the latter study suggest that this discrepancy may, however, lay in both differences in dementia severity as well as methodological differences between these studies (Marner et al., 2012). For a more detailed discussion about the current state of other 5-HT PET- and SPECT radioligands, readers are referred to some excellent reviews (Saulin et al., 2012; Paterson et al., 2013; Billard et al., 2014; Kumar and Mann, 2014). These compounds can be used for evaluation of inclusion- and outcome criteria, but also in dose-occupancy studies.

## Dopaminergic System

The activity of DA, a catecholamine, is mediated through five dopaminergic, metabotropic, G-protein coupled receptors. They are divided into two classes: D_1_-like receptors (D_1_R and D_5_R) and D_2_-like receptors (D_2-4_R), depending on the downstream signaling cascade. Levels of DA are regulated through the activity of the presynaptic DAT, which removes DA from the synaptic cleft to terminate its activity (Mitchell et al., 2011). Dopaminergic neurons are largely located in the midbrain, with many projections throughout the brain (Martorana and Koch, 2014), where they are involved in various neurological processes. Of particular importance here is their role in motivation, cognition, and learning (Xu et al., 2012). Indeed, around 35–40% of AD patients exhibit extrapyramidal symptoms and more than 70% display extensive apathy (Lopez et al., 1997; Mitchell et al., 2011). These symptoms might be explained by the significantly reduced levels of DA and its precursor L-3,4-dihydroxyphenylalanine (L-DOPA) (Storga et al., 1996). Although large involvement of DA in AD is still under debate (Portet et al., 2009; Trillo et al., 2013), several noticeable changes have been documented in the DA receptor density. More specifically, a significant reduced expression of D_1_- and D_2_-like receptors has been documented in the prefrontal cortex and the hippocampus of AD patients (Kemppainen et al., 2003; Kumar and Patel, 2007). Furthermore, alterations of the D_2_R in AD seems positively correlated to BPSD and verbal memory performance (Kemppainen et al., 2003; Tanaka et al., 2003). Conflicting results are, however, reported for the D_2_R density in AD patients (see further) (Piggott et al., 1999; Piggott et al., 2007). Contradictory results are also reported for changes in the DAT levels in AD patients (Murray et al., 1995; Ceravolo et al., 2004). Despite some discrepancy, it is clear that there are important DA changes in the AD brains. Finally, it is to be noted that several *in vivo* experiments on mice, expressing AD like pathology, show that significant behavioral and cognitive deficits can be restored by administering DA reuptake inhibitors and L-DOPA (Ambree et al., 2009; Guzman-Ramos et al., 2012). Aβ oligomers may indeed have an early impact on catecholaminergic transmission (Mura et al., 2010).

There are several modes of interventions toward the failing dopaminergic system in AD patients, mostly used to address apathy (the most common BPSD symptom) and extrapyramidal symptoms. One of many approaches is the use of MAO-B inhibitors, which are discussed in Section “Monoamine Oxidase B” of this review. Another therapeutic method is modulation of the DAT transporter, and thus increasing synaptic DA levels. This was done by methylphenidate and dextroamphetamine in several clinical AD studies (Galynker et al., 1997; Herrmann et al., 2008; Lanctot et al., 2008). Although not selective for the dopaminergic system, an overall improvement was noted in symptoms of apathy on the Apathy Evaluation Scale (AES). There are, however, some concerns about the tolerability of methylphenidate (Padala et al., 2010). Other drugs that are frequently used to treat BPSD symptoms in clinical trials (and routine practice) involving AD patients are the antipsychotic drugs quetiapine, aripiprazole, olanzapine, and risperidone. As FDA- and EMA-approved drugs, these drugs act as partial DA receptor agonist or partial DA receptor antagonist (among often interaction with many other targets). Overall improvement on BPSD symptoms was recorded in a large meta-analysis of the use of antipsychotics in AD patients (Ballard and Waite, 2006). Nevertheless, caution was advised by the FDA with these drugs, as they were associated with an increase in risk of death, and other severe side effects, among elder people with dementia (Ballard and Waite, 2006; De Deyn et al., 2013). Yet another drug, rotigotine, a D_2_R- and D_3_R agonist, was able to show cognitive enhancement on probable AD patients, compared to their age-matched HCs by measuring the cortical excitability and central cholinergic transmission (Martorana et al., 2013).

**FIGURE 9:**
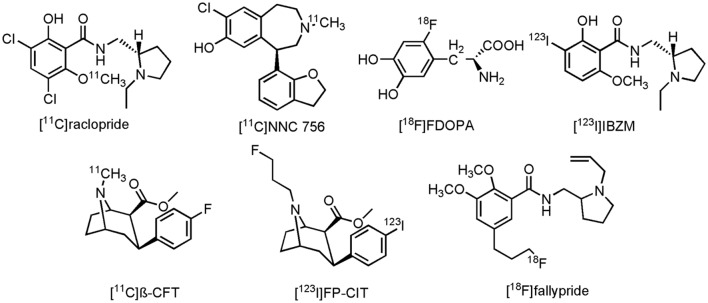
**Structure of dopaminergic PET/SPECT tracers**.

Imaging of the dopaminergic system can be done by a number of PET- and SPECT radioligands. Conflicting results are, however, reported between several clinical studies on AD patients, using different PET- and/or SPECT tracers. In a combined PET study, reduced striatal expression of D_1_R, but not D_2_R was seen with D_2_R antagonist 3,5-dichloro-*N*-{[(2S)-1-ethylpyrrolidin-2-yl]methyl}-2-hydroxy-6-[^11^C]methoxybenzamide([^11^C]raclopride) and D_1_R antagonist (5R)-8-chloro-5-(2,3-dihydro-1-benzofuran-7-yl)-3-[^11^C]methyl-2,3,4,5-tetrahydro-1H-3-benzazepin-7-ol ([^11^C]NNC 756) in AD patients (**Figure 9**), compared to age-matched HCs (Kemppainen et al., 2000). Striatal uptake of 2-amino-3-[2-[^18^F]fluoro-4,5-dihydroxyphenyl]propanoic acid ([^18^F]FDOPA), a fluorinated form of L-DOPA (**Figure 9**), was also unchanged in AD patients, compared to HCs (Tyrrell et al., 1990). Conversely, decreased striatal expression of D_2_R with [^11^C]raclopride was demonstrated in AD patients (with BPSD symptoms) as compared to their HCs (Tanaka et al., 2003). Similar studies, using *N*-{[(2S)-1-ethylpyrrolidin-2-yl]methyl}-2-hydroxy-3-[^123^I]iodo-6-methoxybenzamide ([^123^I]IBZM) (**Figure 9**), a D_2_R antagonist, or methyl (2S,3S)-3-(4-fluorophenyl)-8-[^11^C]methyl-8-azabicyclo[3.2.1]octane-2-carboxylate ([^11^C]β-CFT) (**Figure 9**), a cocaine derivative which binds to DAT, showed, respectively, a reduced expression of D_2_R and a reduced DA reuptake (Pizzolato et al., 1996; Rinne et al., 1998). Reduction of DA reuptake sites, as measured by [^11^C]β-CFT, was hereby positively correlated to the severity of the extrapyramidal symptoms of AD patients, whereas in the study with [^123^I]IBZM, patients did not exhibit any extrapyramidal symptoms. Likewise, a decrease in [^18^F]FDOPA striatal uptake was noticed in another study on AD patients, a decrease which was correlated to the cognitive scores of the AD patients (Itoh et al., 1994). On the other hand, even more confusing, is the fact that in yet another clinical study involving AD patients an increase in D_2_R expression in the striatum was now measured with [^11^C]raclopride (Reeves et al., 2009). Discrepancies between these different studies might, however, be explained by different study populations, and the degree of dementia, since time-dependent dopaminergic receptor changes were also seen in patients with PD (Brooks, 1993). Another role for dopaminergic neuroimaging was displayed by methyl (2S,3S)-8-(3-fluoropropyl)-3-[4-[^123^I]iodophenyl]-8-azabicyclo[3.2.1]octane-2-carboxylate ([^123^I]FP-CIT) (**Figure 9**), an analog of [^11^C]β-CFT. [^123^I]FP-CIT was able to differentiate, with high accuracy, patients with AD, and patients with DLB (Colloby et al., 2008; Spehl et al., 2015). Overall reduced striatal uptake was noticed in both diseases, but lower binding potentials of [^123^I]FP-CIT were reported in DLB than in the case of the AD patients. These scans can greatly improve differential diagnosis between the different neurodegenerative diseases, which often display similar clinical presentations. [^123^I]FP-CIT SPECT scans are already used in clinical routine to distinguish DLB- from AD patients (Spehl et al., 2015). Finally, 5-[3-[^18^F]fluoropropyl]-2,3-dimethoxy-*N*-{[1-(prop-2-en-1-yl)pyrrolidin-2-yl]methyl}benzamide ([^18^F]fallypride) (**Figure 9**), a D_2_R/D_3_R antagonist, could be used to assess the ideal therapeutic window for the use of antipsychotic drugs (Clark-Papasavas et al., 2014), since, as mentioned before, elder people are very sensitive to these drugs. [^18^F]fallypride PET scans could consequently assist in antipsychotic dose-occupancy studies, and thus help to provide an ideal antipsychotic strategy in AD patients with extensive BPSD symptoms.

## Neuroinflammation

### Translocator Protein

Formerly known as PBR, the 18 kDa TSPO, is located on the outer membrane of the mitochondria, predominantly in glial cells. As part of a multimeric complex, which is comprised of a VDAC and an adenine nucleotide carrier (McEnery et al., 1992; Casellas et al., 2002; Cosenza-Nashat et al., 2009), several functions are associated with TSPO (Midzak et al., 2015). They play an essential role in neurosteriod synthesis, by facilitating the transport of cholesterol from the outer to the inner membrane of the mitochondria (Papadopoulos et al., 2006a,b), and hence potentiate the GABAergic neurotransmission through allosteric modulation of the GABA_A_ receptor by neurosteroids (Belelli and Lambert, 2005; Hosie et al., 2006; Rudolph and Mohler, 2006). Furthermore, TSPO may have a crucial function in a variety of cellular processes, such as cell proliferation (Miettinen et al., 1995; Hardwick et al., 1999), mitochondrial respiration (Hirsch et al., 1989) and cell apoptosis (Kugler et al., 2008). In light of TSPO’s association with the pathophysiology of neurodegenerative diseases, it has been well established that part of the neurotoxicity caused by tau and Aβ deposits in AD is induction of a neuroinflammatory response (McGeer and McGeer, 1995; Hoozemans et al., 2011), which triggers the upregulation of TSPO in activated microglia and astrocytes. Moreover, this upregulation clearly correlates with the degree of neuroinflammation, making TSPO a valuable target for drug monitoring (Venneti et al., 2006). Interestingly, in a review by Chua et al. (2014), it was suggested that TSPO ligands may provide effective tools for treatment of AD through activation of neuroprotective pathways of increased expression of astrocytes and microglial cells, since these mechanisms may have a protective phagocytic role in early AD (Morgan et al., 2005). Once a more progressed AD state has been reached, neuroinflammation turns chronic and becomes harmful (Hickman et al., 2008). This view is in contrast with numerous clinical trials, using anti-inflammatory drugs that failed to produce significant improvement in AD patients (Streit, 2010; Venigalla et al., 2015), although this failure may be attributed to a ‘wrong’ stage of the disease when therapy was initiated (Moreira et al., 2006). There are currently no drugs in clinical trials that interact with the TSPO receptor in AD. Drugs that are already described are mainly used for their use against BPSD symptoms (Rupprecht et al., 2009; Owen et al., 2011). TSPO is, however, an important marker for neuroinflammation, which makes it an interesting target for neuroimaging. PET tracers in this class will thus mainly be used to assess inclusion- and outcome criteria in clinical trials with anti-neuroinflammatory drugs in AD.

**FIGURE 10:**
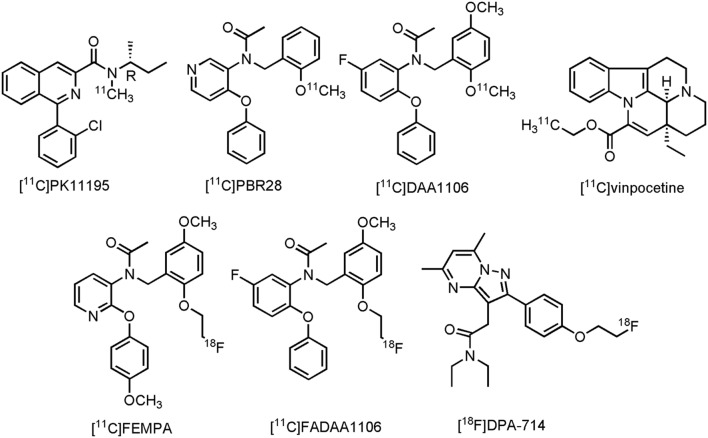
**Structure of TSPO PET tracers**.

The most studied TSPO tracer in patients with CNS disorder is without a doubt *N*-[(2R)-butan-2-yl]-1-(2-chlorophenyl)-*N*-[^11^C]methylisoquinoline-3-carboxamide ([^11^C]PK11195) (**Figure 10**), despite its low specific binding and minimal brain uptake (Damont et al., 2013). Nonetheless, conflicting results are reported with [^11^C]PK11195, but also with several other clinical TPSO tracers, such as *N*-{[2-[^11^C]methoxyphenyl]methyl}-*N*-(4-phenoxypyridin-3-yl)acetamide ([^11^C]PBR28), *N*-(5-fluoro-2-phenoxyphenyl)-*N*-{[2-[^11^C]methoxy-5-methoxyphenyl]methyl}acetamide ([^11^C]DAA1106), (2-[^11^C])ethyl (15S,19S)-15-ethyl-1,11-diazapentacyclo[9.6.2.0^2,7^0^8,18^.0^15,19^]nonadeca-2,4,6,8(18),16-pentaene-17-carboxylate ([^11^C]vinpocetine), *N*-({2-[2-[^18^F]fluor-ethoxy]-5-methoxyphenyl}methyl)-*N*-[2-(4-methoxyphenoxy)pyridin-3-yl]acetamide ([^18^F]FEMPA), *N*-({2-[2-[^18^F]fluoroethoxy]-5-methoxyphenyl}methyl)-*N*-(2-phenoxyphenyl)acetamide ([^18^F]FEDAA1106) and *N,N*-diethyl-2-(2-{4-[2-[^18^F]fluoroethoxy]phenyl}-5,7-dimethylpyrazolo[1,5-a]pyrimidin-3-yl)acetamide ([^18^F]DPA-714) (**Figure 10**). While a majority of clinical trials was able to show significant tracer uptake in at least one brain area in AD patients, several other studies failed to differentiate MCI or even HCs from AD (Varley et al., 2015; Stefaniak and O’Brien, 2016). Apart from low signal-to-noise ratios and low brain uptake of some of these compounds, there are many possible explanations for the discrepancies in TSPO expression, as measured by PET in these clinical trials involving AD patients (Janssen et al., 2016). It is, however, important to realize, as suggested before (Janssen et al., 2016), that many patients exhibit different TSPO expression levels, depending on the specific polymorphism in the TSPO gene, resulting in intersubject variability in the binding affinities of TSPO PET tracers (Owen et al., 2012). Increasing efforts have therefore been focused toward compounds that are insensitive toward TSPO polymorphism, but also toward compounds for other neuroinflammatory targets (such as MAO-B, see Section Monoamine Oxidase B). Furthermore, overexpression of TSPO in both astrocytes and microglial cells make it difficult to differentiate MCI from AD patients (Ekonomou et al., 2015; Janssen et al., 2016). Nevertheless, [^18^F]DPA-714 is currently being used to assess the degree of neuroinflammation in AD patients in two clinical trials (CTI: NCT02377206 and NCT02062099). For a more detailed discussion of the current status of PET development for TSPO, or neuroinflammation in general, readers are referred to several other reviews (Ory et al., 2014; Varley et al., 2015; Janssen et al., 2016).

### Monoamine Oxidase B

**FIGURE 11:**
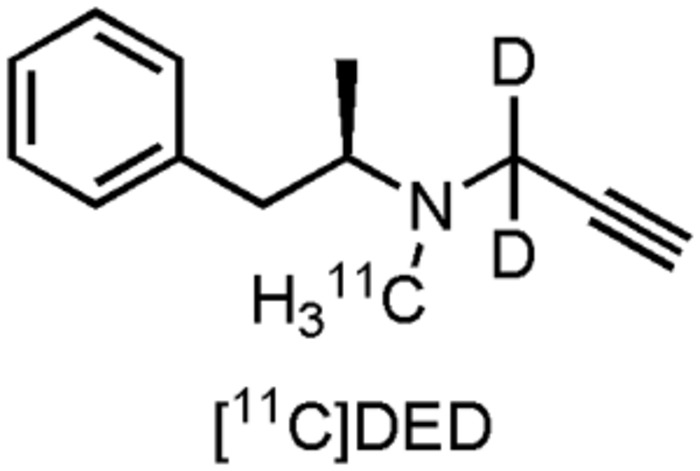
**Structure of MAO-B PET tracer**.

Monoamine oxidases are mitochondrial bound enzymes, as a member of the flavin-containing amine oxidoreductases protein family, in the CNS primarily found in astrocytes. They are responsible for oxidative deamination of monamines of both endogenous and exogenous sources, regulating the physiological activity of neurotransmitters as serotonin, DA, and noradrenaline (Strolin and Dostert, 1989). There are two types of isoforms, MAO-A and MAO-B, which differ in inhibitor sensitivity and substrate selectivity, although there is an overlap to some degree (Bortolato et al., 2008). Increased MAO-B activity has been noted in AD in both brain and blood platelets (Adolfsson et al., 1980; Alexopoulos et al., 1987; Sparks et al., 1991), the severe upregulation in the brain mainly being a consequence of a plaque associated neuroinflammatory response by reactive astrocytes (Jossan et al., 1991; Saura et al., 1994). Furthermore, during their catalytic deamination, MAOs produce neurotoxic byproducts such as hydrogen peroxide, which are one of the main culprits in oxidative stress, contributing to the formation of amyloid plaques (Huang et al., 2012; Zheng et al., 2012). Since MAOs play a key role in the regulation of several important neurotransmitters, cognitive impairment, due to pathological upregulation of MAOs has also been demonstrated (Delumeau et al., 1994). MAO inhibitors may therefore have a significant neuroprotective role in AD. Since MAO-B is the main isoform present in brain (Riederer et al., 1978; Sonsalla and Golbe, 1988) and inhibition of MAO-B proved to be useful as therapeutic approach in PD, focus has mainly been targeted toward MAO-B inhibition in AD (Thomas, 2000). So far, five different drugs have inhibited MAOs in clinical trials involving AD patients (Cai, 2014). Two of them, selegiline (L-deprenyl) and rasagiline (Azilect), irreversible MAO-B selective inhibitors, are established drugs in the treatment of PD, delaying the need for DA replacement therapy (Birkmayer et al., 1977; Lees et al., 1977; Weinreb et al., 2010). While selegiline initially showed promise in clinical trials involving AD patients, demonstrating modest improvements on cognitive and behavioral functions (Filip and Kolibas, 1999), a comprehensive meta-analysis showed no justification for the use of selegiline in the treatment of AD, since there was a lack of overall significant benefit (Birks and Flicker, 2003). The beneficial effect of rasagiline is yet to be evaluated in AD patients. A Phase II proof of concept trial in patients with mild to moderate AD is, however, underway (CTI: NCT02359552). Interestingly, rasagiline formed the basis of two other multi-target drugs, ladogistil (TV3326), and M-30. The former drug is a MAO-B inhibitor and AChE inhibitor, the latter a MAO-A and MAO-B inhibitor (Youdim, 2013). Both compounds are thought to modulate APP expression levels (by stimulating the non-amyloidogenic pathway) and both may have neuroprotective and neurorestorative functions (Riederer et al., 2004; Youdim, 2013). Phase II trials with ladogistil on mild to moderate AD patients have been completed, but results are yet to be published (CTI: NCT01354691). These drugs may hold promise as multi-target approach for treatment of AD, being able to tackle various pathophysiological changes at once (Youdim, 2013). Finally, EVT 301 (RO4477478), a reversible MAO-B inhibitor was evaluated on four AD patients (MMSE score: not specified) in a dose-finding study, using [^11^C]deprenyl-D2 ([^11^C]DED) PET (See further, **Figure 11**) to assess MAO-B occupancy levels. Seven days of treatment resulted hereby in an almost complete dose-occupancy of MAO-B (Hirvonen et al., 2009). No further clinical trials, to our knowledge, have since been performed with this drug.

Only one MAO binding PET tracer has been elevated on AD patients thus far: [^11^C]methyl[(2R)-1-phenylpropan-2-yl][(1,1-^2^H2)prop-2-yn-1-yl]amine ([^11^C]DED), an irreversible MAO-B inhibitor (Hirvonen et al., 2009; Carter et al., 2012; Choo et al., 2014). Increased uptake of [^11^C]DED was observed in patients with MCI (who responded positively to a [^11^C]PIB scan), suggesting that astrocytocis may be an early event in the development of AD. Clinical AD studies with other emerging (fluorine-18 labeled) MAO radiotracers are yet to be published (Ory et al., 2014; Fowler et al., 2015).

## Conclusion

This review demonstrates that there are multiple approaches to be considered when developing disease altering drugs for AD. Important to notice is that each of these pathways is linked to the pathophysiological processes of (often many) other targets, hence a multi-target approach, addressing various pathophysiological changes at once, will be the way forward. Concordant neuroimaging techniques, such as PET and SPECT, could hereby greatly improve therapeutic monitoring, but also significantly aid with the proposal of current inclusion- and outcome criteria in large clinical studies. Moreover, once a disease altering drug has been found, PET/SPECT could eventually be used as standard test to assess and follow up disease progression (Barthel et al., 2015). Still, there are a few important factors to take in consideration. One of them is the need for quantitative PET assessment, especially during evaluation of novel therapies, to allow quantitative and accurate evaluations. Visual inspection or simplified models (such as SUV) are indeed less robust, as they are influenced by several physiological and technical factors (Boellaard, 2009; Tomasi et al., 2012). The other side of the coin is, however, that quantitative PET assessment is very time-consuming, which limits capacity and throughput which are essential in large multi-center trials. Another crucial issue is the loss of BBB function during AD progression, which could greatly affect drug dosage and bioavailability of novel AD therapeutics. One way of monitoring the viability of the BBB is by looking at the P-gp function. Several promising PET candidates are now under development for this purpose (Syvanen and Eriksson, 2013), and a pilot study to assess the P-gp function in AD patients is currently ongoing (ECT: 2013-001724-19). Finally, there is a great need for a thorough preclinical evaluation of the mechanism of action. This could not be better demonstrated than by the rise and fall of dimebon. Initially developed as an antihistaminic drug in the former USSR, dimebon demonstrated significant improvement on the ADAS-cog-, MMSE-, and CIBIC+ scales in a 6-month Phase II trial on AD patients in Russia (Bezprozvanny, 2010). Consequently, a multi-national Phase III trial was launched, but this trial failed to show any significant improvement on mild to moderate AD patients, as compared to placebo (Bezprozvanny, 2010). In a synopsis by Bezprozvanny (Bezprozvanny, 2010), failure was largely attributed to poor understanding of the proper mechanism of action in preclinical studies and the lack of objective biomarkers to assess the true therapeutic response in clinical trials. In the last decade, most clinical trials aiming to find AD combatting drugs ultimately failed to produce (convincing) positive results. Although there may be many explanations for this overall failure, thorough preclinical assessment remains an important factor.

## Author Contributions

LD wrote the manuscript. RV, KVL, AV, and GB reviewed it, corrected it and made suggestions.

## Conflict of Interest Statement

The authors declare that the research was conducted in the absence of any commercial or financial relationships that could be construed as a potential conflict of interest.

## Abbreviations

5-HT: 5-hydroxytryptamine
5-HTRs: 5-HT receptors
5-HTT: 5-HT transporter
α-APP: α-amyloid precursor protein
Aβ: amyloid beta
ACh: acetylcholine
AChE: acetylcholinesterase
AD: Alzheimer’s disease
ADAS-cog: Alzheimer’s disease assessment scale-cognitive subscale
ALA: α-lipoic acid
APP: amyloid precursor protein
BACE1: β-site APP-cleaving enzyme 1
BBB: blood–brain barrier
BPSD: behavioral and psychological symptoms of dementia
BZDs: benzodiazepines
CBD: corticobasal degeneration
ChAT: choline acetyltransferase
CIBIC+: clinician’s interview-based impression of change
CNS: central nervous system
CSF: cerebrospinal fluid
CT: computerized tomography
CTI: clinical trial identifier
D_1_R: dopamine_1_-like receptors
DA: dopamine
DAT: dopamine transporter
DLB: dementia with Lewy bodies
ECT: EudraCT-number
EMA: European medicines agency
FDA: food and drug administration
FTD: frontal temporal dementia
GABA: gamma-amino butyric acid
GSK: glycogen synthase kinase
HCs: healthy controls
mAChR: muscarinic acetylcholine receptor
MAPT: microtubule-associated protein tau gene
MCI: mild cognitive impairment
MMSE: mini-mental state examination
MOAs: monoamine oxidases
MRI: magnetic resonance imaging
nAChRs: nicotinic acetylcholine receptors
NFTs: neurofibrillary tangles
PBR: peripheral benzodiazepine receptor
PET: positron emission tomography
P-gp: permeability glycoprotein
PHFs: paired helical filaments
PSP: progressive supranuclear palsy
p-tau: phosphorylated tau
rCBF: regional cerebral blood flow
SERT: 5-HT reuptake transporter
SP: senile plaques
SPE(C)T: single photon emission (computed) tomography
TSPO: translocator protein
VDAC: voltage dependent anion channel.
